# Impact of External Sources of Indole Acetic Acid and 2,3,5-Triiodobenzoic Acid on Alkaloid Production and Their Relationships with Primary Metabolism and Antioxidant Activity in *Annona emarginata* (Schltdl.) H. Rainer

**DOI:** 10.3390/plants13182637

**Published:** 2024-09-21

**Authors:** Bruna Cavinatti Martin, Ivan De-la-Cruz-Chacón, Carolina Ovile Mimi, Carmen Silvia Fernandes Boaro, Felipe Girotto Campos, Inara Regiane Moreira-Coneglian, Gisela Ferreira

**Affiliations:** 1 Department of Biodiversity and Biostatistics, Institute of Biosciences, São Paulo State University (UNESP), Prof. Dr. Antônio Celso Wagner Zanin Street, 250, Botucatu 18618-689, SP, Brazil; bruna.cavinatti@unesp.br (B.C.M.); carmen.boaro@unesp.br (C.S.F.B.); felipe.girotto@unesp.br (F.G.C.); inara.moreira@unesp.br (I.R.M.-C.); gisela.ferreira@unesp.br (G.F.); 2 Laboratorio de Fisiología y Química Vegetal, Instituto de Ciencias Biológicas, Universidad de Ciencias y Artes de Chiapas, Libramiento Norte-Poniente 1150, Tuxtla Gutiérrez 29039, Chiapas, Mexico; ivan.cruz@unicach.mx

**Keywords:** Annonaceae, IAA, TIBA, plant regulators, nitrogenous secondary metabolites

## Abstract

*Annona emarginata* is a native Brazilian species capable of producing at least ten alkaloids of ecological, agronomic, and pharmacological importance. Some studies have explored the effect of external phytoregulators on the production of alkaloids, including the effect of auxins, which, like alkaloids, derive from the shikimic acid pathway. Thus, this study aimed to evaluate how indole acetic acid (IAA) and its inhibitor 2,3,5-triiodobenzoic acid (TIBA) impact the production of alkaloids and the primary metabolism of *A. emarginata*, which brings advances in the understanding of the mechanisms of alkaloid synthesis and can aid in the bioprospection of molecules of interest in Annonaceae. The design was completely randomized, with three treatments (control, IAA [10^−6^ M] and TIBA [10^−6^ M]) and five collection times (12, 36, 84, 156, and 324 h). The following variables were analyzed: total alkaloids, alkaloid profile, nitrate reductase activity, gas exchange in photosynthesis, chlorophyll *a* fluorescence, sugars, starch, and antioxidant activity. Of the twelve alkaloids analyzed, discretine and xylopine were not detected in the control plants; however, both were detected when IAA was applied (in roots and leaves) and xylopine (in roots) when the inhibitor was applied. The alkaloid asimilobine was not detected with the use of TIBA. Variations in alkaloid concentrations occurred in a punctual manner, without significant variations in photosynthesis and nitrate reductase activity, but with variations in the antioxidant system and sugar concentrations, mainly at 156 h, when the highest alkaloid concentrations were observed with the use of TIBA. It could be concluded that IAA is capable of selectively modulating the production of alkaloids in *A. emarginata*, either due to an external source or by the application of its inhibitor (TIBA).

## 1. Introduction

Specialized metabolism differs in the different groups of species and is dependent on environmental conditions, being directly related to primary metabolism, which is common to all plants and provides precursor molecules for the development of specialized metabolism [1,2,3]. Among specialized metabolites, alkaloids stand out due to their role in the adaptation and survival of plants to various biotic and abiotic factors, constituting a nitrogen reserve and acting in plant defense [4]. In addition, they are bioactive compounds of medical, pharmaceutical, and agronomic interest due to their antifungal, bactericidal, antiprotozoal, cytotoxic, analgesic, and antiplatelet action [5,6,7,8,9]. 

Several abiotic factors, including hormonal balance, are capable of altering primary metabolism and triggering changes in specialized metabolism [10,11]. Among these plant regulators are auxins, which cause changes in primary metabolism, such as increased photosynthetic rates [12,13,14], and are associated with growth regulation, development, plant defense [15,16] and modulation of specialized metabolism [11,17,18,19,20].

Auxin and alkaloids compete for substrate because they share part of the same biosynthetic pathway, the shikimic acid pathway, mainly the tryptophan pathway, which produces indole alkaloids [12,21]. Thus, the application of auxins can interfere with the synthesis of alkaloids in a positive [11] or negative [22] manner, which may be related to the increase in the internal CO_2_ concentration [23] and variations in the synthesis of sugars, which highlights the relationship between primary and specialized metabolism. Such metabolic modifications increase the production of reactive oxygen species, which implies the action of the antioxidant system for stress control [24]. 

The Annonaceae family is of interest in the study of specialized metabolism due to its broad biodiversity and chemodiversity. Alkaloids stand out, with approximately 934 of them having been documented in the family, distributed in 254 species [25]. In Brazil, the Annonaceae family is represented by approximately 389 species belonging to 32 genera [26], among them the genus *Annona*, with 80 species, 24 of which are endemic [27]. The most abundant alkaloids are benzylisoquinolines derived from amino acids phenylalanine and tyrosine; however, indole alkaloids derived from tryptophan have also been reported [25]. 

*Annona emarginata* (Schltdl.) H. Rainer ‘Terra-Fria’ morphotype is native to the South American continent [28], and in Brazil, it has importance in the recovery of degraded areas and in fruit growing, being used as rootstock for the commercial species atemoya (*Annona atemoya* Mabb.) [8,29]. Alkaloids such as liriodenine, anonaine, reticuline, and asimilobine have been identified in this species [8,11,30,31,32]. Some of these alkaloids are of pharmacological [5,6,9] and agricultural interest, with action in the control of phytopathogenic fungi [6,8]. 

Therefore, the diversity and importance of alkaloids in the Annonaceae family stimulate the understanding of factors that may impact their biosynthesis. In this context, considering that there are factors, such as auxins, that act in both primary and specialized metabolism, this work was carried out. The novelty is the study not only of the impact of auxin IAA but also of an auxin transport inhibitor (TIBA) on alkaloid biosynthesis and the possible relationships of these compounds in the availability of resources from primary metabolism [31], in addition to the action on the antioxidant system, indicating variations in the stress levels of processes. Thus, this study aimed to evaluate how indole acetic acid (IAA) and its transport inhibitor (2,3,5-triiodobenzoic acid—TIBA) impact the production of alkaloids and the relationships with primary metabolism (photosynthesis, sugar production), nitrate reductase, and antioxidant activity. 

## 2. Results

Effect on alkaloid production. The highest concentrations of total alkaloids were detected in the roots of *Annona emarginata* regardless of treatment. In the leaves of plants that received IAA, an increase in total alkaloids was observed in relation to the control only in the last evaluation (324 h). In contrast, the application of TIBA, an auxin transport inhibitor, caused an increase in the concentrations of total alkaloids, mainly in the last three evaluations (84, 156, and 324 h). The roots of plants that received IAA or TIBA did not present differences in the amounts of total alkaloids in relation to control in most evaluation times (12, 36, 84, and 324 h). However, at 156 h after treatments, the highest concentration of total alkaloids was observed in roots with the use of TIBA (108.03 µg g^−1^ dry mass), differing from control (78.91 µg g^−1^ dry mass) and IAA (57.18 µg g^−1^ dry mass) (Table 1).

**Figure 1:**
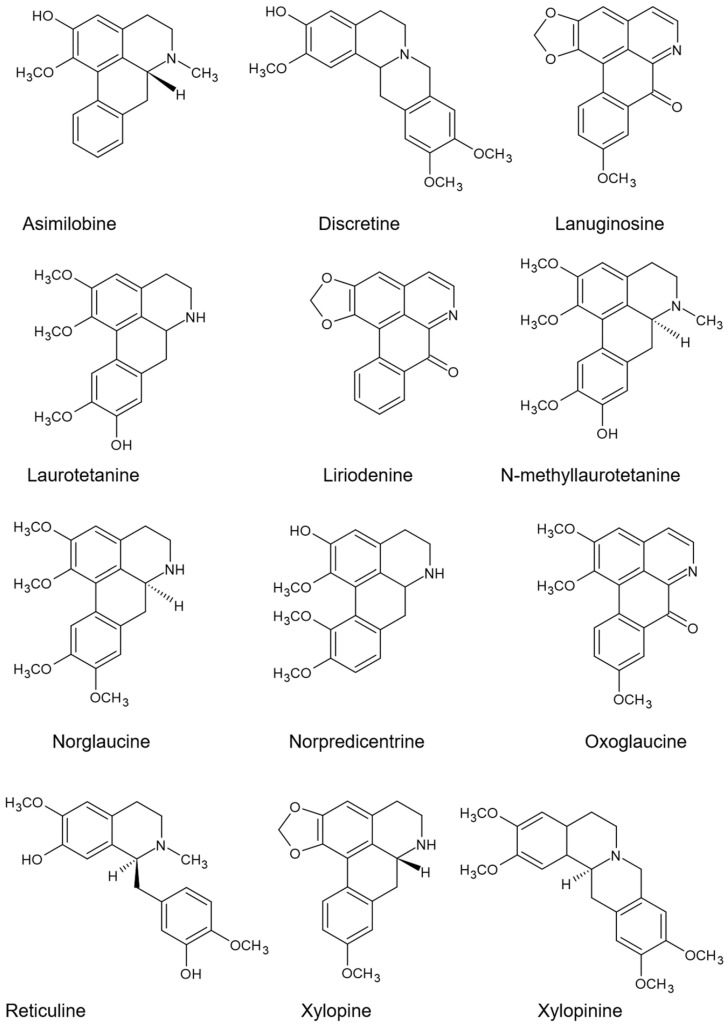
Alkaloids detected in *Annona emarginata* using high-performance liquid chromatograph (UHPLC).

The application of IAA and its transport inhibitor (TIBA) also caused changes in the presence and absence of the 12 investigated alkaloids in *Annona emarginata* (Figure 1), both in leaves and roots (Table 2).

Among the 12 alkaloids under investigation, it was not possible to detect the presence of any of them in the leaves of the control group. However, when IAA was applied, it was possible to detect lanuginosine, liriodenine, and xylopine. Lanuginosine, liriodenine, and *N*-methyllaurotetanine were detected with the application of TIBA, which indicates possible selectivity in relation to the synthesis of Xylopine and *N*-methylllaurotetanine depending on the treatment.

In the roots of control plants, alkaloids discretine and xylopine were not observed, but asimilobine, lanuginosine, laurotetanine, liriodenine, *N*-methyllaurotetanine, norglaucine, norpredicentine, oxoglaucine, reticuline, and xylopinine were observed. On the other hand, when IAA was applied, oxoglaucine and reticuline were not detected. When auxin transport was inhibited, oxoglaucine, reticuline, asimilobine, and discretine were not found.

Regarding the nitrate reductase activity, it was found that there was only a change due to the application of IAA and TIBA in relation to control at 324 h, which demonstrates that nitrogen assimilation was not altered with treatments over time nor at the time of greatest alkaloid synthesis (156 h); therefore, it is not possible to conclude that there was an increase in nitrogen availability for alkaloid synthesis (Table 3).

Effect on primary metabolism. Gas exchange and chlorophyll *a* fluorescence were impacted by treatments in a specific manner. When TIBA (IAA transport inhibitor) was applied at 12 h, 156 h, and 324 h, a reduction in transpiration was observed when compared to plants that received IAA, differing from control only at 324 h (Table 4). Some moments in which reductions in transpiration occurred coincided with greater production of total alkaloids in leaves (156 h and 324 h) and roots (156 h), in addition to lower stomatal conductance at 156 h. However, during these periods of higher concentrations of total alkaloids in plants treated with TIBA, no differences in relation to the potential quantum yield of photosystem II (Fv′/Fm′), specific quantum yield of PSII (yield), photochemical dissipation (qP), and net CO_2_ assimilation rate (Anet) were observed.

As part of the primary metabolism responses to IAA treatment, an increase in the concentration of total sugars was observed in leaves at 36 h and a reduction in roots, and when auxin transport was inhibited, a reduction in total sugars in leaves and an increase in roots was observed at 156 h, which coincided, at this time, with the highest concentrations of total alkaloids in roots and leaves. This increase in the concentration of total sugars in roots may indicate that there was greater translocation to this organ, the site of greatest alkaloid synthesis (Table 5). 

Regarding sucrose in leaves, it was observed that the inhibition of IAA transport caused a reduction in the sucrose concentration in leaves at all collection times, both in relation to IAA application and in relation to control. However, in roots, the lowest sucrose concentrations with the use of TIBA were punctual in relation to IAA and control (12 h and 36 h) (Table 5).

Contrary to what was observed with sucrose in leaves, there was no response pattern regarding the reduction in the concentration of reducing sugars in plants with IAA transport inhibition. What was observed was a punctual reduction at 84 h and an increase in concentration at 324 h (when compared to plants that received IAA, but without differing from control), the time at which the highest concentrations of total alkaloids were observed in leaves treated with TIBA. However, it was possible to observe that the auxin inhibitor caused a reduction in the concentrations of reducing sugars in roots at all evaluation times, while IAA caused an increase in concentrations (Table 5).

Starch concentration in leaves at 84 h and 156 h was high after the application of IAA compared with plants treated with TIBA and control. At the other times (12 h, 36 h and 324 h), the auxin transport inhibitor caused a reduction in starch concentration in leaves. In roots, starch concentration was higher in plants treated with IAA only at 156 h (14.23 µg g^−1^ FW) when compared to plants treated with the inhibitor (9.41 µg g^−1^ FW) (Table 5). This lower value may be related to the increase in total sugars at 156 h and to the synthesis of alkaloids since there is a subsequent reduction in starch, total and reducing sugars in roots between 156 h and 324 h, and an increase in alkaloid concentration. Thus, the lowest sugar concentrations found in roots generally coincide with the periods of highest alkaloid concentrations, especially in roots, which may indicate that sugars were used as a source of energy and/or greater availability of carbon skeletons for the synthesis of alkaloids.

Effect on the antioxidant system. Hydrogen peroxide was not altered in leaves in relation to control, and in roots, IAA caused an increase in hydrogen peroxide concentrations at all evaluation times compared with TIBA inhibitor and control (Table 6). 

There was an increase in lipoperoxide concentrations in leaves at most evaluation times (except for 12 h) when auxin transport was inhibited. In contrast, in roots, the application of IAA caused an increase in lipoperoxide concentration at all evaluation times, except for 324 h, when a drastic reduction was observed, both in relation to TIBA and control. It is noteworthy that at 156 h, the highest lipoperoxide concentration was observed with IAA over time (27.60 nmol g^−1^ MF) in roots, as well as with the use of TIBA (24.04 nmol g^−1^ MF), with differences between both and control (9.45 nmol g^−1^ MF), and at this time, the highest concentration of total alkaloids was observed with the use of TIBA.

Regarding the activity of the antioxidant system, treatments caused a reduction in the superoxide dismutase (SOD) activity in leaves and an increase in roots. At 84 h, differences were observed in roots between treatments, with the highest activity due to the application of IAA (77.8 U µg^−1^ protein), followed by plants that received the transport inhibitor (65.3 U µg^−1^ protein) and control (40.3 U µg^−1^ protein).

No differences were observed in the catalase (CAT) activity in roots and leaves between treatments and control (Table 6).

Regarding the peroxidase (POD) activity in leaves, it was only possible to detect a change between treatments at 324 h, with greater activity due to the application of IAA, with no difference in relation to control. However, in roots, both treatments caused an increase in the POD activity in relation to control, except at 36 h, when only treatment with IAA caused an increase in the POD activity and at 324 h, when treatment with TIBA caused the greatest increase (4.379), both in relation to IAA (1.163) and to control (0.739) (Table 6).

## 3. Discussion

The higher concentrations of total alkaloids found in *Annona emarginata* roots corroborate the reports for this and other species of the genus *Annona* that showed the same pattern of higher concentration of alkaloids in roots than in leaves [30,32,33]. 

Regarding the effect of IAA, the results of this experiment generally demonstrated that there was no significant change in the concentration of total alkaloids over time compared with the control group. However, at 156 h, the increase in total alkaloids with the application of IAA transport inhibitor (TIBA) and the decrease with IAA was noteworthy. These results differ from what was expected since, in a study with *Annona emarginata* and the use of indole butyric acid (IBA), Sousa et al. [11] observed a significant increase in total alkaloids in roots. Similarly, but with species from other botanical families, Mostafa and Abou Alham [34] found that in *Balanites aegyptiaca* leaves, the synthesis of alkaloids increased with the use of IAA. In *Catharanthus roseus*, the foliar application of IAA provides an increase in the production of the indole alkaloid vincristine [17].

On the other hand, Godjin et al. [22] and Pasquali et al. [35] detected a reduction in the concentration of indole alkaloids in the cell culture of *Daucus carota* and *C. Roseus* with the application of auxins 1-naphthaleneacetic acid (NAA), indole acetic acid (IAA) and 2,4-dichlorophenoxyacetic acid (2,4-D). Godjin et al. [22] and Pasquali et al. [35] justified the results due to the lower transcription of the tryptophan decarboxylase gene, which participates in the alkaloid biosynthesis route through the tryptophan pathway and also gives rise to IAA, and the level of these transcripts increased in medium without the auxins tested (IAA, NAA, and 2,4-D). Thus, the fact that indole acetic acid shares the tryptophan pathway in the production of alkaloids [12,21] may justify the lower concentration of alkaloids when there is a higher concentration of auxins. However, this information does not explain what occurs when there is an increase in alkaloids mediated by the application of IAA [17,34], and there is also a need for further studies to clarify the results when there is an increase in alkaloids in Annonaceae [11], which are largely benzylisoquinolinic alkaloids and, therefore, have tyrosine as precursor [25,36]. 

Plant regulators from other groups, such as cytokinins and gibberellins, have also been shown to modulate the synthesis of alkaloids in several *Annona* species. Sousa et al. [37] found that after soaking *Annona cacans* seeds in a solution containing GA_4+7_ + 6-Benzyladenine, the content of total alkaloids and liriodenine doubled in roots of seedlings and in cotyledonary leaves, the number of total alkaloids decreased, and the liriodenine concentration remained unchanged. Silva et al. [38] also observed changes in alkaloids during germination and in *Annona x atemoya* Mabb. cv. ‘Gefner’ seedlings with a reduction in the concentration of total alkaloids after treating seeds with GA_3_ to overcome dormancy.

As observed in this experiment, changes in the detection of different alkaloids with the use of regulators were also observed in other studies, such as that of Sousa et al. [37], where the primary roots (5 cm) of *A. cacans* from seeds treated with a solution containing GA_4+7_ + 6-Benzyladenine showed a decrease in the proportion of alkaloids discretine and reticuline and a reduction in the content of total alkaloids, but the content of liriodenine was not altered. In another study by Sousa et al. [11], it was observed that in *A. emarginata* seedlings, the supply of abscisic acid resulted in the disappearance of the alkaloid laurotetanine when compared to control, while treatments did not cause changes in the content of total alkaloids.

Nitrogen is required for the synthesis of alkaloids since alkaloids are nitrogenous molecules of the specialized metabolism, which is related to the activity of the nitrate reductase enzyme [39]. This is the first enzyme to catalyze the reaction of nitrate to nitrite in the cytosol during the process of nitrate assimilation in organic compounds [14]. The nitrogen atoms of alkaloids come from amino acids, and in most cases, alkaloids maintain in their structure the carbon skeleton of the precursor amino acid [21,40]. In this context, the use of auxin did not result in increased activity of the nitrate reductase enzyme and, therefore, did not promote greater nitrogen availability that would result in greater alkaloid production, as expected. Furthermore, it is not possible to determine how much of the available nitrogen was destined for alkaloid synthesis [39] or for other metabolic processes, such as photosynthesis [41,42]. 

Regarding gas exchange and chlorophyll *a* fluorescence, IAA did not cause variations that could be related to lower photosynthetic efficiency, unlike what was observed by Sousa et al. [11] with the use of another auxin (indole butyric acid—IBA) also in *A. emarginata*. The authors found that IBA promoted a decrease in CO_2_ assimilation that culminated in a reduction in the carboxylation efficiency of the Rubisco enzyme, which was not observed in this experiment. Furthermore, the punctual reduction in stomatal conductance at 156 h and in transpiration at 324 h occurred in response to the use of the IAA inhibitor but without variations in photosynthetic efficiency. Hormones such as auxin and cytokinin inhibit stomatal closure induced by IBA; however, in this experiment, auxin did not cause any variation in stomatal conductance compared with control.

Also, in relation to primary metabolism, variations in carbohydrate levels, especially reductions in concentrations when the highest concentrations of alkaloids (156 and 324 h) are observed with the auxin transport inhibition, are related to the greater availability of carbon skeletons and energy for the synthesis of compounds such as amino acids and, consequently, alkaloids, as suggested by Hikosaka et al. [43] and Nunes-Nessi et al. [44]. The higher concentrations of reducing sugars observed with IAA application corroborate reports by Ono et al. [44], who observed an increase in the concentrations of total and reducing sugars with the application of NAA and IBA. 

When auxin transport was inhibited by TIBA, there was a reduction in sugars, possibly used for the greater synthesis of alkaloids, which corroborates Dudareva et al. [10], who reported that the biosynthesis of specialized metabolites depends on the availability of carbon (C), nitrogen (N), and energy provided by primary metabolism, and as a consequence, the availability of these building blocks affects the concentration of specialized metabolites, demonstrating a high degree of connectivity between primary and specialized metabolisms.

In order to avoid the harmful effect of reactive oxygen species generated by primary metabolism, an increase in enzymatic activity may occur, generating the accumulation of amino acids and the production of alkaloids, with an increase in nitrogen reserves, as proposed by Ghorbanpour [45]. In this context, although IAA has stimulated the highest hydrogen peroxide and lipoperoxide concentrations in most moments in roots, the antioxidant system (especially SOD and POD) acted similarly, both in plants that received IAA and in those that received TIBA and with greater antioxidant activity than that observed with control plants. In other words, there was an increase in the action of the antioxidant system due to the application of both IAA and TIBA and not specifically due to the greater stress caused by free radicals only with the use of IAA in order to guarantee the functionality of the system and avoid cellular damage, which was observed at all evaluation times, corroborating reports by Barbosa et al. [24]. However, the accumulation of amino acids proposed by Ghorbanpour [45] seems to have occurred only at 156 h, when the highest concentrations of total alkaloids were observed with the use of TIBA. At this time, greater stress and greater activity of the antioxidant system were expected; however, values were similar to those observed at other times, indicating that treatments were not effective in sufficiently stressing the system and increasing the synthesis of alkaloids or that the alkaloid synthesis resulted from substrates originating from sources not involved in the antioxidant system, without representing additional stress to the plant. 

## 4. Materials and Methods

### 4.1. Plant Material and Experimental Area

*Annona emarginata* (Schltdl.) H. Rainer (‘terra-fria’ morphotype) seedlings were obtained from the “Seedling Production Center of São Bento do Sapucaí”, CATI (Technical and Integrated Assistance Coordination), municipality of São Bento do Sapucaí—SP. Young 1.5-year-old plants were taken to a greenhouse at the Biodiversity and Biostatistics Department of the Unesp Biosciences Institute of Botucatu, SP (located at 22°53′25″ South, 48°27′19″ West, and an altitude of 800 m a.s.l.; Cwa climate), kept in bags from the original nursery, and irrigated with water daily (sprinkler irrigation) and weekly with 50% Hoagland nutrient solution (manual irrigation) for a period of ten months. 

The greenhouse where the experiment was conducted consists of a metal arch structure covered with 150 μm transparent polyethylene (LDPE, anti-UV) agricultural plastic film and laminated ‘Aluminet’ shade (Polysack^®,^ Nir Yitzhak, Israel) with an automated sprinkler irrigation system. The environmental conditions were determined, where leaf and air temperatures ranged from 24 °C to 29 °C, and relative humidity ranged from 45 to 62%. 

The species was identified by Prof. Renato Melo-Silva, PhD, and samples were deposited at the “Herbário Irina Delanova Gemtchujnicov” BOTU herbarium, São Paulo State University, Campus of Botucatu—SP, under code 33120.

### 4.2. Experimental Design

The study was conducted in a completely randomized experimental design with a 3 × 5 factorial scheme with five replicates of one plant per treatment, three treatments (auxin, auxin transport inhibitor, and control), and five collection times (12 h, 36 h, 84 h, 156 h, and 324 h after the last application of treatments).

Indole acetic acid (IAA) at a concentration of 10^−6^ M was used as the source of auxin, and 2,3,5-triiodobenzoic acid (TIBA) at a concentration of 10^−6^ M was used as an auxin transport inhibitor. These IAA and TIBA concentrations were defined according to a previous experiment carried out by Sousa et al. [11]. IAA and TIBA were purchased from Sigma-Aldrich^®^ Brasil Ltda, Sao Paulo, Brazil.

### 4.3. Application of Indole Acetic Acid and TIBA Transport Inhibitor

Three foliar applications were performed at 48 h intervals using a pressurized carbon dioxide backpack sprayer with a fan nozzle and pressure of 276 bar. The non-ionic Haiten^®^ (Ningbo, China) adhesive was used as the spreading agent at a rate of 1 mL/10 L of solution, as recommended by the manufacturer, and was also used with water in the control treatment. 

### 4.4. Quantitative and Qualitative Analysis of Alkaloid Extracts

For the extraction of alkaloids, leaf and root material from the five replicates of each treatment (auxin, auxin inhibitor, and control) was used at each collection time (12 h, 36 h, 84 h, 156 h, and 324 h after the last application of treatments). To obtain the total alkaloid extracts, leaf and root samples (1.5 g) were dried in an oven with forced ventilation (40 °C) until reaching constant dry mass and then ground using the mechanical mill. Extraction was performed using the acid-base method [6,46]. Alkaloid extracts were kept in the dark until the total alkaloid content was determined using a UV-visible spectrophotometer (single beam—model UV-M51—BEL Engineering^®^, Monza, Italy) at 254 nm. Liriodenine was used with a calibration curve of 1–100 mg mL^−1^ to prepare the standard curve (y = 0.0881x − 0.0112, R^2^ = 0.9949) since it is one of the most widely distributed alkaloids among species of the Annonaceae family [47]. Liriodenine standard was provided by Iván De-la-Cruz-Chacón (coauthor). The methods for the isolation and data identification of liriodenine were reported in De-la-Cruz-Chacón and González-Esquinca [46].

The presence of alkaloids was detected using a high-performance liquid chromatograph (UHPLCfocused ThermoFisher-Scientific, Waltham, MA, USA) with gradient pump and UV-VIS detector using a C18 reversed-phase column (150 × 4.6 mm and particle diameter of 5 μm) at 30 °C. The mobile phase was water (with trifluoroacetic acid pH 3.5) and methanol at a ratio of 30:70, with a flow rate of 1 mL/min. Detection was performed in UV at 280 nm [46]. The identification of alkaloids asimilobine, discretine, lanuginosine, laurotetanine, liriodenine, *N*-methyllaurotetanine, norglaucine, norpredicentine, oxoglaucine, reticuline, xylopine, and xylopinine was also carried out by the comparison to standards provided by Emmanoel Vilaça Costa and Jackson Roberto Guedes da Silva Almeida (spectrometric data were previously reported in Sousa et al. [37]).

### 4.5. Nitrate Reductase Activity 

The activity of the nitrate reductase enzyme was determined according to the methodology proposed by Mulder et al. [48], which is based on the production of NO_2−_ during the incubation of 1 g of leaf cuttings in the presence of NO_3−_. Leaf samples (200 mg) from the five replicates of each treatment (auxin, auxin inhibitor, and control) were used to perform the analysis at each collection time (12 h, 36 h, 84 h, 156 h, and 324 h after the last application of treatments) in duplicate.

### 4.6. Gas Exchange and Chlorophyll a Fluorescence

Evaluations were performed at 12 h, 36 h, 156 h, and 324 h after the last application of plant growth regulators in the period from 09:00 a.m. to 11:00 a.m. on the third or fourth fully expanded leaves in five replicates per treatment, except for 84 h after the application of treatments, as it was raining, which did not allow the equipment to function properly. An open photosynthesis system equipment with CO_2_ and water vapor analyzer by infrared radiation (Infra Red Gas Analyzer—IRGA, model GSF 3000- Heinz Walz GmbH, Effeltrich, Germany) was used. The gas exchange variables analyzed were net CO_2_ assimilation rate (A, μmol CO_2_ m^−2^ s^−1^), transpiration rate (E water vapor m^−2^ s^−1^), stomatal conductance (gs, mol m^−2^ s^−1^), and internal CO_2_ concentration in the substomatal chamber (Ci, µmol m^−2^ s^−1^), adopting the average natural light conditions (DFFFA) of each evaluation time incident inside the cultivation environment, on average 867 PAR µmol m^−2^ s^−1^. From these data, it was possible to calculate the carboxylation efficiency (A/Ci) (µmol CO_2_ m^−2^ s^−1^/µmol m^−2^ s^−1^). Regarding fluorescence, the potential quantum yield of photosystem II (Fv/Fm), the electron transport rate (ETR), and the photochemical extinction coefficient (qP) were analyzed [49]. 

### 4.7. Carbohydrate Concentration

Total soluble sugars were extracted from leaf (100 mg) and root (100 mg) material obtained from five replicates of each treatment (auxin, auxin inhibitor, and control) at each collection time (12 h, 36 h, 84 h, 156 h, and 324 h after the last application of treatments), according to Garcia et al. [50], and to determine the concentration of total soluble sugars, the method proposed by to Morris [51] was used. Starch was extracted according to Clegg [52] and quantified according to Yemm and Folkes [53]. For reducing sugars, the methodology established by Miller [54] was used, and for sucrose, the methodology was determined by Passos [55].

### 4.8. Antioxidant Activity and Reactive Oxygen Species

Leaf and root samples (100 mg) from the five replicates of each treatment (auxin, auxin inhibitor, and control) at each collection time (12 h, 36 h, 84 h, 156 h, and 324 h after the last application of treatments) were immediately immersed in liquid nitrogen and subsequently macerated in a mortar with the aid of liquid nitrogen and stored in a freezer at −20 °C. The methodology proposed by Kar and Misshra [56] was followed to obtain the enzymatic extract. To determine the specific activity of antioxidant enzymes, it was necessary to evaluate the protein concentration using the Bradford methodology [57]. Catalase activity (CAT, EC 1.11.1.6) was determined according to Peixoto et al. [58], peroxidase activity (POD, EC. 1.11.1.7) according to Teisseire and Guy [59], and superoxide dismutase activity (SOD, EC 1.15.1.1) according to the Beauchamp and Fridovich technique [60]. 

Regarding reactive oxygen species, hydrogen peroxide content was determined according to the method of Alexieva et al. [61], and lipid peroxidation was determined according to the methodology proposed by Heath and Paker [62].

### 4.9. Data Analysis

Data were submitted to normality and homogeneity of variance tests. The results of quantification analyses of indole acetic acid, gas exchange, and chlorophyll *a* fluorescence were submitted to one-way ANOVA analysis of variance, and the means were compared using Tukey’s test (*p* ≤ 0.05).

The results of quantification analyses of total alkaloids and liriodenine, the activity of the nitrate reductase enzyme, carbohydrate concentration (total sugars, reducing sugars, starch, and sucrose), and the activity of antioxidant enzymes (SOD, POD, and CAT) were submitted to two-way ANOVA, and means were compared using Tukey’s test (*p* ≤ 0.05).

## 5. Conclusions

Indole acetic acid promotes selective variations in alkaloid production in the leaves and roots of young *Annona emarginata* plants. In leaves, with the use of IAA, it was possible to detect lanuginosine, liriodenine, and xylopine, and when the IAA transport was inhibited by TIBA, the synthesis of xylopine was inhibited, and the production of *N*-methyllaurotetanine was induced, while lanuginosine and liriodenine remained present. In roots, 10 alkaloids were detected, and IAA promoted the appearance of discretine and xylopine (which did not appear in control) and inhibited the synthesis of oxoglaucine and reticuline. With the application of TIBA (auxin transport inhibition), asimilobine, discretine, oxoglaucine, and reticuline were not observed, but xylopine (absent in control) and the other alkaloids analyzed were observed. IAA did not cause significant variations in photosynthesis over a long period of evaluation in a way that impacts the production of alkaloids. However, when the IAA transport was inhibited by TIBA, there was a punctual increase in the production of sugars and starch, which resulted in higher alkaloid concentrations. Greater stress was observed with the application of IAA and TIBA and greater activity of the antioxidant system; however, no increase in stress was observed with the increase in the production of total alkaloids. The activity of nitrate reductase also did not increase with the application of IAA and IBA, demonstrating that nitrogen redistribution occurred in order to increase alkaloid production. 

## Figures and Tables

**Table 1:** Concentration of total alkaloids (µg g^−1^ dry mass) in the leaves and roots of *Annona emarginata* in each treatment (Control, indole acetic acid—IAA and its transport inhibitor [2,3,5-triiodobenzoic acid—TIBA]), at different collection times (12 h, 36 h, 84 h, 156 h, and 324 h after the application of treatments).

Total Alkaloids (µg g^−1^ Dry Mass)					
	12 h	36 h	84 h	156 h	324 h
Leaves					
Control	2.69 ± 0.23 Cb*^1^	9.49 ± 1.46 Aa	5.81 ± 0.72 Bb	3.12 ± 0.8 Cb	0.917 ± 0.31 Dc
IAA	3.19 ± 0.94 Aab	2.83 ± 0.21 Ac	0.90 ± 0.08 Bc	3.22 ± 0.77 Ab	2.32 ± 0.43 Ab
TIBA	4.31 ± 1.63 Ba	7.43 ± 1.97 Bb	9.67 ± 2.01 Aba	10.99 ± 2.17 Aa	11.55 ± 3.04 Aa
Treatments *p*: 0.0001; f: 68.219; Hours: *p* 0.0001; f: 37.935; Treatments×Hours *p*: 0.0001; f: 74.589					
Roots					
Control	66.07 ± 2.78 Aa	66.62 ± 3.27 Aab	76.87 ± 4.11 Aa	78.91 ± 3.65 Ab	77.44 ± 4.27 Aab
IAA	55.43 ± 3.05 Aa	37.73 ± 2.55 Bb	72.39 ± 4.14 Aa	57.18 ± 2.87 Ac	68.16 ± 3.32 Ab
TIBA	71.02 ± 3.67 Ba	70.83 ± 2.81 Ba	74.69 ± 5.66 Ba	108.03 ± 9.12 Aa	91.50 ± 6.96 ABa
Treatments *p*: 0.0001; f: 28.246; Hours: *p* 0.0001; f: 10.394; Treatments×Hours *p*: 0.0002; f: 3.455					

*^1^ Results are presented as mean ± standard deviation. Means followed by the same uppercase letter in the row and lowercase letter in the column do not show significant differences according to the Tukey test.

**Table 2:** Presence (x) and absence (-) of alkaloids in leaves and roots of *Annona emarginata* after the application of treatments (Control, indole acetic acid IAA and its transport inhibitor (2,3,5-triiodobenzoic acid—TIBA)) at five collection times (12 h, 36 h, 84 h, 156 h, and 324 h after the application of treatments).

Leaves (L)/Roots (R)					
	12 h (L/R)	36 h (L/R)	84 h (L/R)	156 h (L/R)	324 h (L/R)
Control					
Asimilobine	-/-	-/x	-/x	-/-	-/-
Discretine	-/-	-/-	-/-	-/-	-/-
Lanuginosine	-/x	-/x	-/x	-/x	-/x
Laurotetanine	-/-	-/x	-/x	-/x	-/x
Liriodenine	-/x	-/x	-/x	-/x	-/x
*N*-methyllaurotetanine	-/x	-/x	-/x	-/x	-/x
Norglaucine	-/x	-/x	-/x	-/x	-/x
Norpredicentine	-/-	-/x	-/x	-/-	-/x
Oxoglaucine	-/-	-/x	-/-	-/-	-/-
Reticuline	-/x	-/x	-/x	-/x	-/x
Xylopine	-/-	-/-	-/-	-/-	-/-
Xylopinine	-/x	-/x	-/x	-/x	-/x
IAA					
Asimilobine	-/-	-/-	-/-	-/x	-/-
Discretine	-/-	-/-	-/-	-/x	-/-
Lanuginosine	-/x	-/x	x/x	x/x	-/x
Laurotetanine	-/-	-/x	-/-	-/x	-/-
Liriodenine	-/x	x/x	x/x	x/x	-/x
*N*-methyllaurotetanine	-/x	-/x	-/x	-/x	-/x
Norglaucine	-/x	-/x	-/x	-/x	-/x
Norpredicentine	-/-	-/x	-/-	-/-	-/-
Oxoglaucine	-/-	-/-	-/-	-/-	-/-
Reticuline	-/-	-/-	-/-	-/-	-/-
Xylopine	-/-	x/x	-/x	-/-	-/x
Xylopinine	-/x	-/x	-/-	-/x	-/-
TIBA					
Asimilobine	-/-	-/-	-/-	-/-	-/-
Discretine	-/-	-/-	-/-	-/-	-/-
Lanuginosine	-/x	-/x	x/x	x/x	-/x
Laurotetanine	-/-	-/x	-/-	-/x	-/x
Liriodenine	-/x	x/x	x/x	x/x	-/x
*N*-methyllaurotetanine	-/x	x/x	-/x	x/x	-/x
Norglaucine	-/x	-/x	-/x	-/x	-/x
Norpredicentine	-/-	-/-	-/-	-/-	-/x
Oxoglaucine	-/-	-/-	-/-	-/-	-/-
Reticuline	-/-	-/-	-/-	-/-	-/-
Xylopine	-/-	-/x	-/x	-/x	-/x
Xylopinine	-/-	-/x	-/-	-/x	-/-

“-“ symbolizes the absence and “X” the presence of each alkaloid in the row by time in the column.

**Table 3:** Nitrate reductase activity (µg N-NO_2_ g of MF^−1^ h^−1^) in *Annona emarginata* leaves at each collection time after the application of treatments (Control, indole acetic acid IAA and its transport inhibitor [2,3,5-triiodobenzoic acid—TIBA]).

Nitrate Reductase (µg N-NO_2_ g de MF^−1^ h^−1^)	12 h	36 h	84 h	156 h	324 h
Control	3.1665 ± 0.195 Ba*¹	2.9165 ± 0.108 Ba	2.6959 ± 0.102 Ba	3.2196 ± 0.220 Ba	4.1528 ± 0.385 Aa
IAA	3.2182 ± 0.034 Aa	2.8571 ± 0.133 Aa	3.1928 ± 0.140 Aa	2.8261 ± 0.204 Aa	3.4460 ± 0.200 Ab
TIBA	3.4396 ± 0.244 Aa	3.0666 ± 0.148 Aa	3.0943 ± 0.152 Aa	3.0779 ± 0.109 Aa	3.2224 ± 0.112 Ab
Treatments *p*: 0.5742; f: 0.56; Hours: *p* 0.0002; f: 6.66; Treatments×Hours *p*: 0.014; f: 2.66					

*^1^ Results are presented as mean ± standard deviation. Means followed by the same uppercase letter in the row and lowercase letter in the column do not present significant differences between them by the Tukey test.

**Table 4:** Gas exchange and chlorophyll *a* fluorescence evaluated after the application of treatments (IAA, TIBA, and control): potential quantum yield of photosystem II (Fv′/Fm′), specific quantum yield of PSII (yield), photochemical dissipation (qP), transpiration (mmol water vapor m^−2^ s^−1^), net CO_2_ assimilation rate [Anet] (μmol CO_2_ m^−2^ s^−1^), and stomatal conductance (gs) (mol m^−2^ s^−1^).

Treatments	Fv′/Fm′	Yield	qP	Transpiration	Anet	Gs
	12 h					
Control	0.423 ± 0.089 AB*^1^	0.063 ± 0.008 A	0.156 ± 0.021 A	0.794 ± 0.102 AB	6.01 ± 1.97 AB	34.9 ± 5.9 AB
IAA	0.467 ± 0.076 A	0.081 ± 0.13 A	0.179 ± 0.067 A	1.281 ± 0.121 A	8.62 ± 2.01 A	60.8 ± 4.3 A
TIBA	0.317 ± 0.059 B	0.067 ± 0.007 A	0.2090.055 A	0.587 ± 0.097 B	3.99 ± 1.02 B	24.2 ± 4.6 B
	*p*: 0.0001 f: 22.019	*p*: 0.0001 f: 30.118	*p*: 0.0001 f: 20.342	*p*: 0.0002 f: 25.556	*p*: 0.0001 f: 20.488	*p*: 0.0002 f: 10.733
	36 h					
Control	0.434 ± 0.88 A	0.083 ± 0.01 A	0.194 ± 0.032 A	1.040 ± 0.2 A	8.28 ± 2.97 A	46.2 ± 6 A
IAA	0.365 ± 0.37 A	0.084 ± 0.008 A	0.225 ± 0.027 A	0.872 ± 0.186 A	7.94 ± 2.03 AB	39.5 ± 5.1 A
TIBA	0.358 ± 0.69 A	0.064 ± 0.005 A	0.187 ± 0.054 A	0.577 ± 0.099 A	5.24 ± 0.82 B	24.6 ± 4.5 A
	*p*: 0.0001 f: 13.441	*p*: 0.0003 f: 23.643	*p*: 0.0002 f: 24.559	*p*: 0.0005 f: 31.497	*p*: 0.0001 f: 22.362	*p*: 0.0005 f: 25.461
	156 h					
Control	0.509 ± 0.48 A	0.177 ± 0.025 A	0.352 ± 0.087 A	1.622 ± 0.182 AB	6.49 ± 1.8 A	63.7 ± 4.8 A
IAA	0.500 ± 0.67 A	0.180 ± 0.068 A	0.366 ± 0.075 A	1.819 ± 0.177 A	7.00 ± 1.81 A	72.5 ± 7.1 A
TIBA	0.444 ± 0.55 A	0.183 ± 0.045 A	0.411 ± 0.054 A	1.259 ± 0.098 B	6.78 ± 2 A	49.1 ± 6.7 B
	*p*: 0.0001 f: 10.231	*p*: 0.0001 f: 20.226	*p*: 0.0005 f: 26.768	*p*: 0.0002 f: 10.211	*p*: 0.0005 f: 20.389	*p*: 0.0001 f: 42.435
	324 h					
Control	0.450 ± 0.066 A	0.068 ± 0.012 A	0.159 ± 0.054 A	0.903 ± 0.134 A	7.59 ± 2.26 A	55.9 ± 5.4 A
IAA	0.474 ± 0.78 A	0.081 ± 0.009 A	0.172 ± 0.047 A	1.008 ± 0.122 A	9.24 ± 3.23 A	59.9 ± 4.9 A
TIBA	0.418 ± 0.098 A	0.071 ± 0.023 A	0.171 ± 0.074 A	0.694 ± 0.078 B	6.56 ± 1.87 A	45.6 ± 4.3 A
	*p*: 0.0005 f: 21.367	*p*: 0.0001 f: 13.793	*p*: 0.0002 f: 10.245	*p*: 0.0001 f: 18.742	*p*: 0.0003 f: 30.277	*p*: 0.0003 f: 61.223

*^1^ Results are presented as mean ± standard deviation. Means followed by the same uppercase letter in the row and lowercase letter in the column do not show significant differences between them according to the Tukey test. IAA: indole acetic acid. TIBA (2,3,5-triiodobenzoic acid) is an inhibitor of IAA transport.

**Table 5:** Concentration of total sugars (µg g^−1^ FW), reducing sugars (µg g^−1^ FW), starch (µg g^−1^ FW), and sucrose (µg g^−1^ FW) in the leaves and roots of *Annona emarginata* after the application of treatments with plant growth regulators at each collection time.

**Total sugars**					
Leaves					
Treatments	12 h	36 h	84 h	156 h	324 h
Control	1268.9 ± 322.1 Aa*¹	1305.3 ± 245.2 Ab	1307.5 ± 245.5 Aa	1356.1 ± 343.8 Aa	1468.7 ± 321.5 Aa
IAA	1272.1 ± 298.3 Ba	1830.5 ± 356.6 Aa	1411.8 ± 365.4 Ba	1431.5 ± 278.9 Ba	1383.0 ± 289.2 Ba
TIBA	1191.3 ± 336.5 Ba	1279.5 ± 254.7 Ab	1486.2 ± 321.3 Aa	1206.5 ± 311.4 Bb	1568.7 ± 231.2 Aa
Treatments *p*: 0.0001; f: 31.22; Hours: *p* 0.0002; f: 23.11; Treatments×Hours *p*: 0.0002; f: 12.67					
Roots					
Treatments	12 h	36 h	84 h	156 h	324 h
Control	269.8 ± 32.1 Cb	466.6 ± 52 Aa	333.7 ± 37.8 Ba	377.0 ± 44.5 Bb	362.7 ± 47.6 Ba
IAA	355.8 ± 21.7 Aa	349.7 ± 39.7 Ab	304.9 ± 40.5 Aa	379.3 ± 39.1 Ab	356.5 ± 36.7 Aa
TIBA	305.2 ± 45 Bab	426.1 ± 65.2 Aba	360.3 ± 32.7 Ba	478.3 ± 58.9 Aa	354.6 ± 34.1 ABa
Treatments *p*: 0.0001; f: 21.99; Hours: *p* 0.0001; f: 10.03; Treatments×Hours *p*: 0.0005; f: 32.45					
**Reducing sugars**					
Leaves					
Treatments	12 h	36 h	84 h	156 h	324 h
Control	1.15 ± 0.06 Aa	0.92 ± 0.07 Bb	0.94 ± 0.08 Ba	0.82 ± 0.1 Ba	0.79 ± 0.05 Bab
IAA	0.75 ± 0.02 Bb	1.06 ± 0.12 Aa	1.05 ± 0.09 Aa	0.77 ± 0.08 Ba	0.71 ± 0.07 Bb
TIBA	0.84 ± 0.03 Ab	0.84 ± 0.05 Ab	0.79 ± 0.03 Ab	0.85 ± 0.06 Aa	0.91 ± 0.1 Aa
Treatments *p*: 0.0001; f: 13.92; Hours: *p* 0.0001; f: 23.79; Treatments×Hours *p*: 0.0003; f: 12.86					
Roots					
Treatments	12 h	36 h	84 h	156 h	324 h
Control	0.772 ± 0.12 Ab	0.780 ± 0.14 Ab	0.712 ± 0.23 Ab	0.728 ± 0.21 Ac	0.692 ± 0.14 Ab
IAA	1.191 ± 0.2 Aa	1.159 ± 0.21 Aba	1.085 ± 0.3 ABa	1.165 ± 0.32 ABa	1.06 ± 0.22 Ba
TIBA	0.531 ± 0.16 Bc	0.538 ± 0.1 Bc	0.598 ± 0.19 Bc	0.834 ± 0.31 Ab	0.582 ± 0.29 Bc
Treatments *p*: 0.0002; f: 25.36; Hours: *p* 0.0001; f: 17.64; Treatments×Hours *p*: 0.0001; f: 21.77					
**Starch**					
Leaves					
Treatments	12 h	36 h	84 h	156 h	324 h
Control	39.3 ± 5.3 Aba	43.4 ± 6.1 Aa	24.6 ± 2.1 Bb	35.4 ± 5.1 ABb	46.4 ± 4.8 Aa
IAA	31.6 ± 4.6 Bb	29.5 ± 4.2 Bb	46.2 ± 4.7 Aa	47.4 ± 6.2 Aa	22.5 ± 3.7 Cc
TIBA	22.1 ± 3.7 Cc	19.2 ± 2.6 Cc	28.4 ± 2.9 Bb	37.2 ± 3.3 Ab	31.3 ± 3.9 Bb
Treatments *p*: 0.0003; f: 27.37; Hours: *p* 0.0001; f: 10.55; Treatments×Hours *p*: 0.0002; f: 18.87					
Roots					
Treatments	12 h	36 h	84 h	156 h	324 h
Control	7.61 ± 3.5 Aa	9.71 ± 5.2 Ab	7.43 ± 3.7 Aa	9.23 ± 3.6 Ab	8.78 ± 2.8 Aa
IAA	7.78 ± 4.1 Ba	14.87 ± 6.1 Aa	7.94 ± 3.2 Ba	14.23 ± 5.7 Aa	8.36 ± 3.1 Ba
TIBA	8.29 ± 3.9 Ba	13.49 ± 5.9 Aa	9.03 ± 4.1 Ba	9.41 ± 4.3 Bb	6.62 ± 2.2 Ca
Treatments *p*: 0.0002; f: 40.33; Hours: *p* 0.0001; f: 22.49; Treatments×Hours *p*: 0.0003; f: 12.28					
**Sucrose**					
Leaves					
Treatments	12 h	36 h	84 h	156 h	324 h
Control	33.57 ± 4.3 Aa	34.82 ± 3.8 Aa	34.31 ± 4.5 Aa	35.46 ± 3.1 Aa	33.59 ± 2.9 Aa
IAA	33.71 ± 3.9 Aa	33.33 ± 3.2 Aa	33.11 ± 5.1 Aa	34.70 ± 4.2 Aa	33.41 ± 3.5 Aa
TIBA	29.12 ± 2.8 Ab	29.48 ± 2.4 Ab	29.26 ± 1.9 Ab	29.75 ± 3 Ab	30.47 ± 2.6 Ab
Treatments *p*: 0.0005; f: 30.29; Hours: *p* 0.0005; f: 34.78; Treatments×Hours *p*: 0.0002; f: 9.54					
Roots					
Treatments	12 h	36 h	84 h	156 h	324 h
Control	25.7 ± 4.2 Ba	29.1 ± 2.9 Aa	26.7 ± 3.7 ABa	26.7 ± 4 ABa	24.0 ± 2.8 Ba
IAA	26.6 ± 4.8 Aa	27.2 ± 2.9 Aab	27.1 ± 2.8 Aa	26.6 ± 3.5 Aa	25.4 ± 2.2 Aa
TIBA	23.1 ± 3.9 Bb	25.3 ± 3.2 ABb	25.0 ± 3.1 ABa	26.3 ± 5.1 Aa	25.5 ± 3 Aba
Treatments *p*: 0.0001; f: 32.3; Hours: *p* 0.0001; f: 14.57; Treatments×Hours *p*: 0.0002; f: 19.71					

*^1^ Results are presented as mean ± standard deviation. Means followed by the same capital letter in the row and lowercase letter in the column do not present significant differences between them by the Tukey test. IAA: indole acetic acid. TIBA (2,3,5-triiodobenzoic acid) IAA transport inhibitor.

**Table 6:** Analysis of antioxidant enzymes peroxidase (POD—µmol purpurogallin min^−1^ mg prot^−1^), catalase (CAT—μKat μg ^−1^ protein) and superoxide dismutase (SOD—U µg^−1^ protein) and reactive oxygen species: hydrogen peroxide (µmol H_2_O_2_ g^−1^ MF) and lipoperoxide (nmol g^−1^ MF) in leaves and roots of *Annona emarginata* after the application of treatments with regulators at each collection time.

**Hydrogen Peroxide**					
Leaves					
Treatments	12 h	36 h	84 h	156 h	324 h
Control	1.39 ± 0.29 Aa*^1^	1.37 ± 0.4 Aa	1.24 ± 0.3 Aab	1.19 ± 0.2 Aa	1.16 ± 0.2 Aa
IAA	1.21 ± 0.3 Aa	1.26 ± 0.3 Aa	1.30 ± 0.3 Aa	1.04 ± 0.3 Aa	1.08 ± 0.3 Aa
TIBA	1.28 ± 0.35 Aa	1.19 ± 0.4 ABa	1.03 ± 0.2 ABb	1.02 ± 0.2 ABa	0.97 ± 0.25 Ba
Treatments *p*: 0.0003; f: 27.322; Hours: *p* 0.0005; f: 13.224; Treatments×Hours *p*: 0.0001; f: 35.887					
Roots					
Treatments	12 h	36 h	84 h	156 h	324 h
Control	0.120 ± 0.03 Ab	0.134 ± 0.24 Ab	0.143 ± 0.23 Ab	0.138 ± 0.06 Ab	0.152 ± 0.024 Ab
IAA	0.218 ± 0.07 Aa	0.230 ± 0.56 Aa	0.417 ± 0.49 Aa	0.252 ± 0.4 Aa	0.174 ± 0.05 Aa
TIBA	0.088 ± 0.02 Ab	0.139 ± 0.45 Ab	0.090 ± 0.02 Ab	0.090 ± 0.002 Ab	0.084 ± 0.019 Ab
Treatments *p*: 0.0002; f: 21.134; Hours: *p* 0.0001; f: 29.624; Treatments×Hours *p*: 0.0002; f: 19.728					
**Lipoperoxide**					
Leaves					
Treatments	12 h	36 h	84 h	156 h	324 h
Control	11.4 ± 4.5 Aa	14.1 ± 5.6 Ab	9.9 ± 3 ABb	8.6 ± 2.7 Bb	8.3 ± 3.2 Bb
IAA	10.7 ± 3.9 Aa	12.7 ± 4.3 Ab	12.1 ± 4.2 Ab	10.5 ± 3 Aab	10.4 ± 3.8 Ab
TIBA	12.6 ± 5 Ca	22.8 ± 5.8 Aa	17.8 ± 5.1 Ba	12.7 ± 3.5 Ca	13.7 ± 4.2 Ca
Treatments *p*: 0.0001; f: 9.934; Hours: *p* 0.0001; f: 18.998; Treatments×Hours *p*: 0.0002; f: 30.609					
Roots					
Treatments	12 h	36 h	84 h	156 h	324 h
Control	6.33 ± 2 Bb	11.33 ± 3.1 Ab	9.44 ± 3.1 Ab	9.45 ± 3.2 Ac	8.80 ± 3 Aa
IAA	10.78 ± 3.2 Ca	19.43 ± 5 Ba	12.37 ± 4 Ca	27.60 ± 7.1 Aa	6.40 ± 1.7 Db
TIBA	7.72 ± 2.2 Bb	8.97 ± 2.9 Bc	8.93 ± 2.5 Bb	24.04 ± 7 Ab	10.23 ± 4.1 Ba
Treatments *p*: 0.0001; f: 18.293; Hours: *p* 0.0001; f: 14.576; Treatments×Hours *p*: 0.0002; f: 24.255					
**Superoxide dismutase (SOD)**					
Leaves					
Treatments	12 h	36 h	84 h	156 h	324 h
Control	176.0 ± 21 Ba	217.0 ± 32.1 Ba	490.6 ± 27.8 Aa	155.7 ± 13.6 Ba	119.3 ± 17.5 Ba
IAA	0.42 ± 0.12 Ab	0.16 ± 0.007 Ab	0.12 ± 0.003 Ab	0.06 ± 0.002 Ab	0.05 ± 0.001 Ab
TIBA	0.05 ± 0.007 Ab	0.04 ± 0.001 Ab	0.04 ± 0.009 Ab	0.04 ± 0.002 Ab	0.03 ± 0.001 Ab
Treatments *p*: 0.0001; f: 45.233; Hours: *p* 0.0003; f: 31.724; Treatments×Hours *p*: 0.0002; f: 20.776					
Roots					
Treatments	12 h	36 h	84 h	156 h	324 h
Control	39.8 ± 6.2 Ab	44.3 ± 6.1 Ab	40.3 ± 5.3 Ac	48.0 ± 6 Ab	48.7 ± 5.8 Ab
IAA	63.6 ± 8 Ba	76.1 ± 8.9 Aa	77.8 ± 7.2 Aa	76.9 ± 8.2 Aa	76.5 ± 8.6 Aa
TIBA	61.4 ± 8.5 Ba	68.7 ± 7.5 Ba	65.3 ± 7.1 Bb	80.8 ± 7.9 Aa	71.8 ± 8.1 ABa
Treatments *p*: 0.0001; f: 10.444; Hours: *p* 0.0001; f: 20.411; Treatments×Hours *p*: 0.0001; f: 5.981					
**Catalase (CAT)**					
Leaves					
Treatments	12 h	36 h	84 h	156 h	324 h
Control	0.0031 ± 0.001 Aa	0.0026 ± 0.001 Aa	0.0028 ± 0.0008 Aa	0.0011 ± 0.0001 Aa	0.0028 ± 0.0012 Aa
IAA	0.0014 ± 0.0007 Aa	0.0014 ± 0.001 Aa	0.0009 ± 0.0002 Aa	0.0008 ± 0.0002 Aa	0.0025 ± 0.0009 Aa
TIBA	0.0025 ± 0.001 Aa	0.0013 ± 0.001 Aa	0.0022 ± 0.001 Aa	0.0012 ± 0.0004 Aa	0.0019 ± 0.0007 Aa
Treatments *p*: 0.0005; f: 10.903; Hours: *p* 0.0001; f: 27.774; Treatments×Hours *p*: 0.0003; f: 12.356					
Roots					
Treatments	12 h	36 h	84 h	156 h	324 h
Control	0.00125 ± 0.00007 Aa	0.0010 ± 0.0002 Ab	0.0014 ± 0.0003 Aa	0.0011 ± 0.0005 Aa	0.0021 ± 0.0006 Aa
IAA	0.00249 ± 0.0012 Aa	0.0050 ± 0.001 Aa	0.0026 ± 0.0009 Aa	0.0024 ± 0.0008 Aa	0.0029 ± 0.0009 Aa
TIBA	0.00209 ± 0.0011 Aa	0.0015 ± 0.0003 Aab	0.0029 ± 0.0002 Aa	0.0015 ± 0.0003 Aa	0.0010 ± 0.0002 Aa
Treatments *p*: 0.0457; f: 3.25; Hours: *p* 0.8982; f: 0.27; Treatments×Hours *p*: 0.5585; f: 0.86					
**Peroxidase**					
Leaves					
Treatments	12 h	36 h	84 h	156 h	324 h
Control	0.420 ± 0.13 Ba	0.499 ± 0.2 ABa	0.275 ± 0.012 Ba	0.307 ± 0.15 Ba	1.460 ± 0.4 Aab
IAA	0.149 ± 0.05 Ba	0.870 ± 0.34 Ba	0.798 ± 0.3 Ba	0.525 ± 0.24 Ba	2.008 ± 0.6 Aa
TIBA	0.301 ± 0.23 Aa	0.373 ± 0.21 Aa	0.595 ± 0.22 Aa	0.335 ± 0.11 Aa	0.831 ± 0.3 Ab
Treatments *p*: 0.0001; f: 30.093; Hours: *p* 0.0001; f: 10.677; Treatments×Hours *p*: 0.0002; f: 21.589					
Roots					
Treatments	12 h	36 h	84 h	156 h	324 h
Control	0.222 ± 0.13 Ab	0.558 ± 0.2 Ab	0.481 ± 0.023 Ab	0.518 ± 0.16 Ab	0.739 ± 0.27 Ac
IAA	0.847 ± 0.24 Ba	1.439 ± 0.4 Aa	1.210 ± 0.48 ABa	1.149 ± 0.45 ABa	1.163 ± 0.5 Ab
TIBA	1.024 ± 0.42 Ba	0.840 ± 0.03 Bb	1.225 ± 0.5 Ba	1.108 ± 0.43 Ba	4.379 ± 1.1 Aa
Treatments *p*: 0.0001; f: 30.233; Hours: *p* 0.0001; f: 10.665; Treatments×Hours *p*: 0.0002; f: 10.801					

*^1^ Results are presented as mean ± standard deviation. Means followed by the same capital letter in the row and lowercase in the column do not present significant differences between them according to the Tukey test. IAA: indole acetic acid. TIBA (2,3,5-triiodobenzoic acid) IAA transport inhibitor.

## Data Availability

The original contributions presented in the study are included in the article, further inquiries can be directed to the corresponding author.
